# A Survey of Symbiotic Radio: Methodologies, Applications, and Future Directions

**DOI:** 10.3390/s23052511

**Published:** 2023-02-24

**Authors:** Muhammad Bilal Janjua, Hüseyin Arslan

**Affiliations:** Department of Electrical and Electronics Engineering, Istanbul Medipol University, 34810 Istanbul, Turkey

**Keywords:** ambient backscatter communication, coexistence, passive Internet-of-Things, sixth generation, spectrum sharing, symbiotic communication, symbiotic radio

## Abstract

The sixth generation (6G) wireless technology aims to achieve global connectivity with environmentally sustainable networks to improve the overall quality of life. The driving force behind these networks is the rapid evolution of the Internet of Things (IoT), which has led to a proliferation of wireless applications across various domains through the massive deployment of IoT devices. The major challenge is to support these devices with limited radio spectrum and energy-efficient communication. Symbiotic radio (SRad) technology is a promising solution that enables cooperative resource-sharing among radio systems through symbiotic relationships. By fostering mutualistic and competitive resource sharing, SRad technology enables the achievement of both common and individual objectives among the different systems. It is a cutting-edge approach that allows for the creation of new paradigms and efficient resource sharing and management. In this article, we present a detailed survey of SRad with the goal of offering valuable insights for future research and applications. To achieve this, we delve into the fundamental concepts of SRad technology, including radio symbiosis and its symbiotic relationships for coexistence and resource sharing among radio systems. We then review the state-of-the-art methodologies in-depth and introduce potential applications. Finally, we identify and discuss the open challenges and future research directions in this field.

## 1. Introduction

6G technology has the potential to revolutionize communication networks by connecting the world through sustainable means, with the goal of improving quality of life [1]. The rapid advancement of IoT is driving this change, as it is projected that by 2030 the number of connected devices will reach 125 billion [2], exacerbating the issue of spectrum scarcity. In addition, the majority of IoT devices are energy-constrained and designed to have a long lifespan. One of the major power-consuming components of these devices is the communication radio, which requires energy-intensive components such as amplifiers and oscillators. To address the challenges of spectrum scarcity and power consumption in IoT devices, a new class of IoT known as passive IoT has been proposed in 3GPP technical studies [3]. The goal of this new class of IoT is to design and implement ultra-low power passive communication technologies to enhance spectrum efficiency and support the deployment of large numbers of IoT devices and sensors, using the existing wireless infrastructure.

BackCom is an emerging candidate for ultra-low power wireless communication, in which backscatter technology is used to modulate and reflect incident signals transmitted by RF source for information transfer [4]. This approach eliminates the need for energy-intensive and costly electronic components for RF signal generation at the device, thus congruent with the objective of passive IoT to reduce power consumption and enhance the longevity of IoT devices. The signal required for BackCom can be sourced from a dedicated signal generator or an ambient RF source such as the base station of cellular, TV, or radio broadcast systems. While the use of a dedicated signal generator has been widely adopted in RFID technology due to its low power and low-cost advantages at the device level, the deployment costs of such systems can be a major limitation to scalability. This is because as the network grows, more dedicated signal sources are required, leading to increased costs [5].

The utilization of ambient RF signals for BackCom has been the subject of extensive investigation as a means of surmounting the limitations of RFID and establishing an alternative technology that is known as ABCm [6]. Interestingly, it eliminates the need for dedicated RF source deployment by enabling BackCom to use the signals from wireless systems in the surrounding environment. This approach not only reduces the cost and complexity of deployment but also allows for greater scalability and flexibility in terms of the available signal sources, which can be sourced from existing infrastructure, rather than requiring dedicated RF sources [7]. For instance, in [8], researchers have demonstrated information transfer between two BD by re-modulating and backscattering the incident signals emitted by a nearby TV station. While ABCm has the potential to enhance overall radio resource utilization and spectral efficiency, its performance is often hindered by interference from strong direct-path signals at the receiver [9]. To address this issue, various approaches have been proposed, such as shifting the incident signal to a different frequency band [10,11,12,13], exploiting the cyclical features of the source signal [14,15,16,17], and utilizing multiple antenna receivers for interference suppression [18,19,20]. However, it should be noted that these methods tend to come with a trade-off, such as increased spectrum utilization or receiver complexity, which can limit the practical applications of ABCm. To overcome these challenges, one potential solution is to enable cooperative coexistence between passive devices and existing wireless systems, through radio resource sharing. This approach can help to mitigate the impact of increased spectrum utilization and receiver complexity, while also enabling more efficient use of available resources and increasing the scalability of the system.

In traditional coexistence methods, devices from different systems independently share radio resources by transmitting their own RF signals. However, in the case of ABCm-assisted passive IoT, devices depend on the signals emitted by other devices and RF sources in the environment. This type of resource sharing is similar to symbiotic relationships in biology, where different species form associations to share resources such as food, shelter, and protection. These associations, known as symbiosis, can be either obligatory or facultative, depending on the degree of mutual dependence between the organisms [21]. In facultative symbiosis, two organisms can survive independently but also choose to share resources. In contrast, in obligatory symbiosis, one organism is dependent on the other and cannot survive independently. In both cases, the resource sharing between the organisms can range from mutualism, where both organisms benefit, to competition, where each organism primarily benefits itself [22]. The concept of symbiosis can also be applied to the coexistence of passive devices and existing wireless systems. In this scenario, a SRad paradigm has been introduced, where the wireless systems are designed to support the passive devices with their radio resources for communication while still performing their primary functions. This allows for a mutually beneficial relationship, where both the passive devices and the existing wireless systems can thrive [23]. To the best of the author’s knowledge, no previous survey has presented a systematic summary of SRad systems. This survey provides a comprehensive overview of SRad systems for coexistence and resource sharing of passive and active radio systems. The survey covers fundamental concepts and state-of-the-art methodologies and examines the integration of advanced technologies such as MIMO, RIS, full-duplex communication, and active load. It also presents the potential applications of SRad in 6G wireless networks for various industries, including healthcare, transportation, agriculture, manufacturing, and logistics. The survey also identifies the important challenges in developing and implementing SRad for passive IoT and highlights key areas for future research in this field.

## 2. Organization of Paper

**Figure 1:**
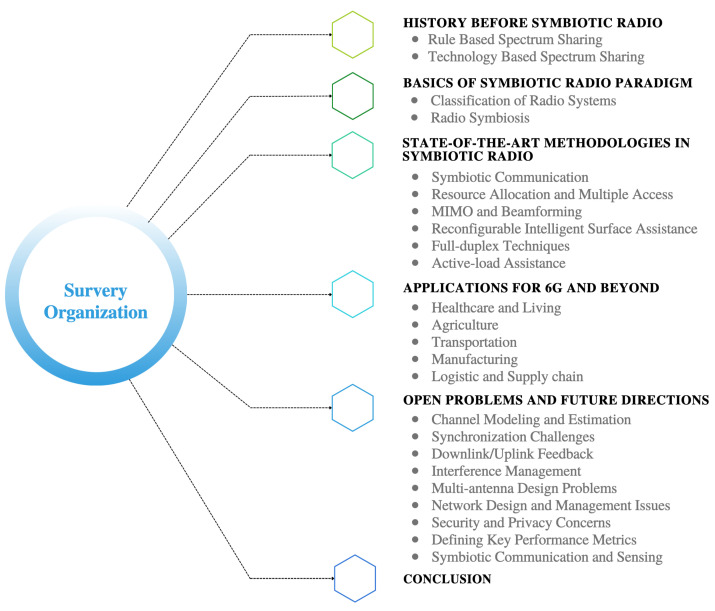
The organization of the sections in this article.

The structure of this survey is outlined in Figure 1. History before SRad is described in Section 3. In Section 4, we provide an introduction to the fundamental concepts of SRad paradigms and radio symbiosis. The state-of-the-art methodologies in SRad are examined in Section 5. In Section 6, we explore the potential applications of SRad systems. The critical challenges and future directions for research and development of SRad are discussed in Section 7. Finally, we present our conclusion in Section 8.

## 3. History before Symbiotic Radio

**Figure 2:**
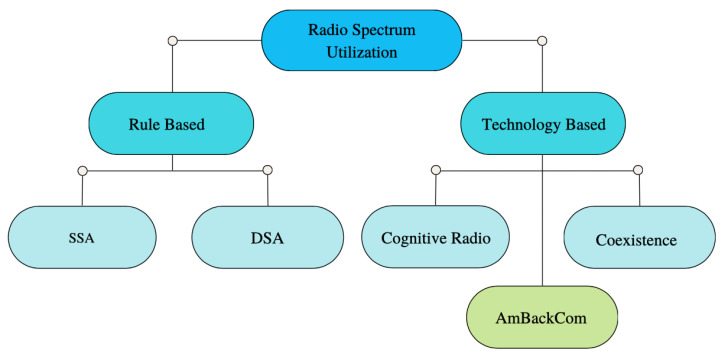
Conventional methods for radio spectrum utilization.

The evolution of IoT has been significantly impacted by the issue of spectrum scarcity or the lack of available radio spectrum. However, this problem has been present since the early days of radio transmissions, as exemplified by Guglielmo Marconi’s transatlantic transmission over a century ago, which occupied the entire radio spectrum at the time. As the number of people and companies using radio transmissions increased, debilitating interference became a significant concern [24]. Addressing the issue of spectrum scarcity requires a multi-faceted approach, which can be looked at from two perspectives: rule-based spectrum allocation and access and technological solutions aimed at improving spectrum utilization. Based on these perspectives, researchers and engineers have developed various mechanisms to improve the existing radio spectrum utilization, which are presented in Figure 2.

### 3.1. Rule Based Spectrum Sharing

In general, two approaches are considered for spectrum access, i.e., SSA and DSA. The SSA is categorized into licensed, rule-based, or unlicensed access. The exclusive licensing strategy has served cellular networks until the recent development of IoT and the proliferation of wireless devices, which needs the spectrum in abundance more than ever before [25]. In rule-based access, certain conditions must be satisfied for accessing the radio spectrum, such as fee payment, transmit power levels, and unoccupied channels. Lastly, the license-exempt or unlicensed access is initiated by the United States FCC in the ISM bands, which allows every technology to access the spectrum with equal rights under some basic regulations [26]. On the other hand, DSA provides more flexible spectrum usage methods than SSA to improve spectrum efficiency. It preserves the rights of exclusive use for licensees and permits them to trade and sell spectrum. Besides, dynamic spectrum allocation can be performed to assign spectrum based on the traffic statistics of a particular service to enhance spectrum efficiency [27]. These regulations and allocations may vary between countries according to their legislative bodies, geo-locations, operating conditions, and technological developments; however, the common objective is to achieve the optimum utilization and to overcome the shortfall of spectrum resources [28].

### 3.2. Technology Based Spectrum Sharing

From a technological perspective, different approaches have been considered to accommodate the upcoming surge of IoT devices and high spectrum demands, including allocation of new bands (i.e., mmWave and THz) [29], re-allocation of legacy licensed spectrum bands [30], the coexistence of wireless technologies [31,32,33], and CR [34,35,36,37]. The first two approaches require either innovations, technological enhancements, transfer of property rights, or clearing the existing users, which are exorbitant and time-consuming [30]. The coexistence of technologies is considered more convenient to overcome the spectrum scarcity issue, where different wireless systems can share and access the resources with minimal interference. Several spectrum-sharing approaches have been proposed in the literature for the coexistence of systems such as WiFi and TV in TVWS [38,39,40], integrated non-terrestrial and satellite [41], WiFi and LTE in the unlicensed 5-GHz band [42], narrow band IoT and LTE [43], and communication and Radar [44]. The coexistence of different wireless systems has been a successful approach for efficient spectrum utilization; however, resource sharing among different wireless systems brings major interference issues.

During the last two decades, CR has been extensively explored for enhancing the spectrum efficiency of under-utilized bands. CR technology enables efficient use of the limited radio spectrum by allowing secondary users to access unused spectrum bands licensed to primary users in a non-interfering manner [45]. Secondary users in CR technology use four primary spectrum sharing techniques: overlay, underlay, interweave, and opportunistic. Secondary users require to vacate the frequency band in overlay spectrum sharing when a primary user is detected. In contrast, secondary users transmit at a power level lower than the primary user’s signal power threshold in underlay sharing. Interweave spectrum sharing involves secondary users identifying unoccupied frequency bands and transmitting only when the primary user is not using the spectrum. Finally, opportunistic spectrum sharing allows secondary users to access the spectrum when primary users are not using it but requires them to vacate the band as soon as a primary user is detected. The goal of spectrum sharing in CR is to enable more efficient use of the radio spectrum while protecting primary users from interference [38]. These techniques are essential for the effective deployment of CR technology, which promises to create a more flexible and efficient wireless network in the future. However, CR systems can create unavoidable interference to the primary systems if not operated under defined regulations. Due to the interference concerns of CR, their adoption in real-time applications is limited so far [22].

To support low-power devices, ABCm has become popular due to its ability to improve overall spectral efficiency without additional energy or infrastructure costs, in which the communication device utilizes the resources of existing wireless systems in a passive manner [8]. Unlike earlier co-existing systems, dedicated infrastructure and resource allocation are not required for ABCm systems [7]. One of the main advantages of this technology lies in its passive way of spectrum sharing without causing significant interference, which can enhance the spectral efficiency of the existing wireless system. Furthermore, it can work with the current spectrum-sharing approaches. However, the backscattering system faces strong interference because of the absence of coordination with the existing wireless systems. As a result, the performance of ABCm is limited by interference [9]. Although coexistence mechanisms have been mentioned sporadically in the literature [26,38,39,40,41,42,43,44,46,47,48,49], we need a general framework that can define other possible means of coexistence and coordination between active and passive radio systems.

The SRad framework facilitates coexistence and coordination among wireless systems to enable efficient resource sharing, combining the benefits of both CR and ABCm [48]. SRad enables the cooperative sharing of spectrum resources among devices from different systems while mitigating interference issues through coordinated access. Compared to CR, the secondary system in SRad employs backscattering communication, resulting in minimal interference to the primary system. Additionally, SRad allows for infrastructure sharing, where multiple systems can share a common transmitter or receiver, thus achieving reliable backscattering communication and overcoming direct link interference issues encountered in ABCm. Overall, SRad offers a flexible and efficient approach to resource sharing and coexistence, making it a promising technology for future wireless communication systems.

## 4. Basics of Symbiotic Radio Paradigm

In this section, we present a general classification of radio systems and revisit the traditional resource-sharing methods with their limitations. We then describe the origination of radio symbiosis and its two variants. Lastly, we discuss the resource-sharing methods based on symbiotic relationships in the SRad system.

### 4.1. Classification of Radio Systems

The radio systems can be categorized into active and passive types based on the RF signal generation requirement at the device. Active radio systems generate an RF signal at the device for information transfer and extract the information from the transmitted signal at the receiver. In a passive radio system, there is no need for active RF signal generation at the device. Instead, external signals are reflected/backscattered for information transfer. The receiver processes the reflected/backscattered signals for information extraction.

**Figure 3:**
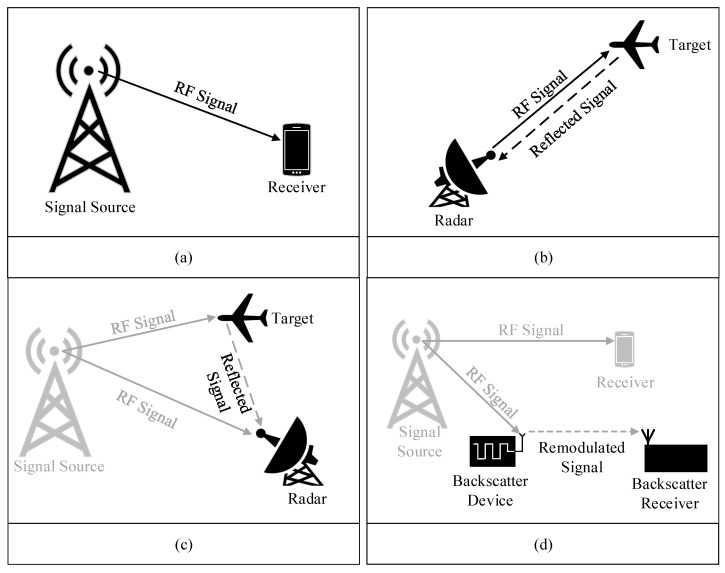
Classification of radio systems: (**a**) active communication system, (**b**) active Radar/ Sensing system, (**c**) passive Radar system, (**d**) passive communication system.

A simple active radio system consists of two parts; a transmitter equipped with a dedicated signal source and a receiver. The transmitter sends the information by transmitting a radio signal in the air, where the receiver detects the signal and extracts the information. The radio signal can be an information-modulated signal or a continuous wave signal depending on whether the system is for communication or Radar. Figure 3a shows an active communication system with a transmitter sending a signal to the receiver for information transfer. On the other hand, Figure 3b shows an active Radar system, which contains a transceiver that radiates the radio wave in the air to detect the presence of the target and its related information. The common examples of active communication systems are TV, cellular, Bluetooth, and WiFi while active Radar systems are mapping, earth monitoring, and navigation.

The use of passive radios based on the backscattering principle can be dated back to the development of passive Radar technology used in World War II. A passive radio reflector was mounted on the allied aircraft to backscatter the signal transmitted by the home Radar with better illumination than enemy aircraft [50]. Afterward, Harry Stockman proposed the idea of reflected power communication in 1948, which contributed to the development of RFID technologies [51]. However, a completely passive RFID system that is operated and controlled by the illuminated signals was demonstrated by Alfred Koelle in 1975 [52]. Since then, RFID technology has been widely used in various fields such as medical, business, logistics, and so on [53]. Passive radio systems are used for communication in simple low-power devices and sensors and for Radar in military applications. A passive Radar system is shown in Figure 3c, where the receiver utilizes the radio waves radiated by ambient radio systems for target detection instead of generating a signal [54]. On the other hand, in a passive communication such as BackCom, a BD or tag re-modulates the ambient RF signals to send the information to a receiver as shown in Figure 3d. The ambient RF signal source can be any wireless system including a tiny Bluetooth device, a large satellite base station placed in space, and every system in between [55,56,57,58].

### 4.2. Radio Symbiosis

A natural ecosystem consists of two components, namely biotic and abiotic. Biotic components include all living organisms such as animals, plants, bacteria, etc., and abiotic components refer to non-living physical resources that affect the ecosystem, such as water, soil, minerals, etc. Multiple biotic components live together in a biological ecosystem and have different associations while sharing physical resources for survival or facultative. In 1879, a pathologist named Anton de Bary coined the term symbiosis, which means living together for interspecific associations or interactions among dissimilar organisms [59]. He defines symbiosis as “interspecific associations, or symbioses, a phenomenon in which two different species of organisms depend on each other for food, shelter, or protection”. The organism that takes part in the symbiosis is called a symbiont. Symbiotic relationships define how two or more symbionts interact for resource sharing. The symbiosis can be facultative and obligatory as per the dependence of the symbionts on each other. If one or two symbionts depend entirely on each other and cannot survive without symbiosis, this type is called obligatory symbiosis. On the other hand, if both symbionts can survive independently and engage in symbiosis, this type of symbiosis is termed facultative or optional symbiosis. Symbionts can have different symbiotic relationships to achieve common or individual objectives. The possible symbiotic relationships include but are not limited to mutualism, commensalism, parasitism, and competition [60].

**Figure 4:**
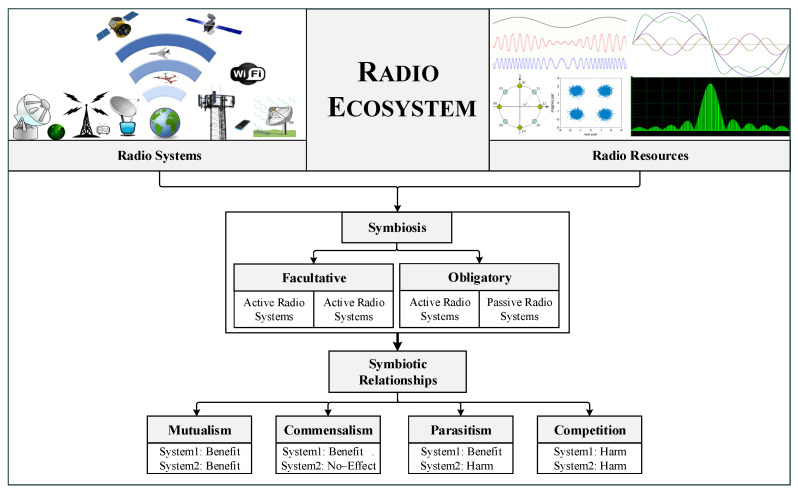
Symbiosis in the radio ecosystem.

The radio ecosystem is somehow not different than the biological ecosystem. The radio resources and systems are similar to the abiotic and biotic components. The radio systems utilize the radio resources such as time, frequency, and space, to perform their functionalities, e.g., communication, and Radar. The radio ecosystem evolves rapidly with the proliferation of wireless applications. Yet, the radio resources, particularly the frequency spectrum, are limited and insufficient to accommodate the needs of radio systems. Different radio systems can engage in symbiosis to meet the radio resource requirements for intelligent radio resource sharing. Radio symbiosis is analogous to biological symbiosis, where two or more radio systems have inter-specific associations and interactions for sharing resources. The radio system that is developed from symbiosis is known as an SRad system. In addition, radio symbiosis can be obligatory or facultative according to the dependencies of radio systems. In facultative symbiosis, both systems are active and share the resources, even though they do not depend on each other e.g., LTE and WiFi. On the other hand, in obligatory symbiosis, one radio system is passive and the other is active and both share the same resources, where the passive radio system depends on the resources of the active radio system e.g., passive IoT and WiFi [6,58]. The radio symbiosis enables the coordination among the radio systems for intelligent resource utilization and to achieve common or individual objectives from symbiosis in an SRad system [48]. There are several types of symbiotic relationships that can be developed between the different radio systems and users for sharing resources [22,48]. Figure 4 illustrates a flow diagram of SRad system from radio symbiosis to symbiotic relationships in the radio ecosystem. The possible symbiotic relationships include:

- Mutualism relationship: In this type of relationship, both systems work together to improve the overall performance of the network, resulting in a positive impact on both systems.
- Commensal relationship: In this type of relationship, one system maximizes its data rate without considering the performance of the other.
- Parasitic relationship: In this type of relationship, one system transmits at the same rate as the other to achieve maximum data rate, but at the expense of the transmission rate of the other system.
- Competition relationship: In this type of relationship, both systems try to gain the maximum transmission rate by competing for resources, which can lead to a reduction in performance for both systems.

Interestingly, in each symbiotic relationship, one radio system receives the benefit from symbiosis but the other system either gets benefit or harm. Nonetheless, the overall resource utilization efficiency is increased. As the radio systems provide a wide range of functionalities, the SRad systems can be designed from radio systems that provide the same radio service e.g., communication only, or radio systems that provide different services e.g, Radar and communication. An example of former SRad is SCm, while symbiotic RADAR is an example of the latter. In SCm, different radio systems share the radio resources to perform communication tasks [22]. However, radio systems share the radio resources to perform Radar/sensing and communication functions in symbiotic RADAR [61,62,63]. In this survey, we will focus on SCm in SRad with obligatory symbiosis.

## 5. State-of-the-Art Methodologies in Symbiotic Radio

### 5.1. Symbiotic Communication

**Figure 5:**
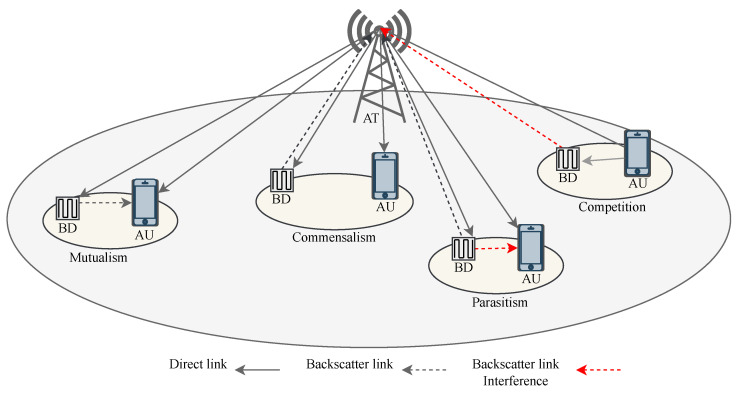
SRad system with different symbiotic relationships between BD and AU for resource sharing.

In SCm, relevant radio systems establish symbiotic relationships to enable communication. Unlike the conventional communication methods, SCm allows the radio systems to cooperate and share the resources intelligently in an SRad system. Figure 5 shows the SRad system for enabling SCm between active and passive radio systems. The active radio system comprises an AT and an AU that engages in conventional active radio communication. Meanwhile, a passive radio system with a BD sends data through BackCom over the signals transmitted by AT. These two systems communicate through SCm in obligatory symbiosis, characterized by efficient resource and service exchange. In this way, the BD performs energy harvesting and information transfer tasks without requiring additional infrastructure, energy, and spectrum [23]. In return, the BD can provide services such as data relaying depending on the different symbiotic relationships.

**Figure 6:**
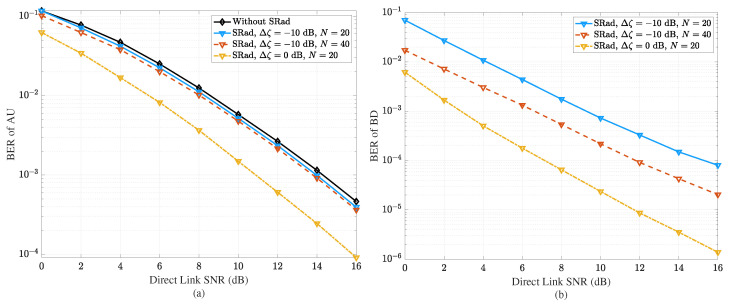
SRad performance in terms of (**a**) BER of AU with QPSK modulation. (**b**) BER of BD with BPSK modulation. Δζ represents the ratio of SNR between the backscattering link and direct link, and the duration of one BD symbol is equal to N times of AU symbol.

In a mutualistic relationship, the transmission rate of the backscattered signal is selected to be significantly lower than the active signal, thus avoiding interference to the AU while providing additional multi-path gain [64]. Even in a commensal relationship, the BD modulation rate is low, but the backscattered signal is weak and does not add multi-path gain or interference to the AU. Here, the BD benefits from utilizing the signal of the AU without providing any benefit or harm. On the other hand, in a parasitic relationship, the BD backscatters the signal at a high data rate, creating interference to the AU and thereby improving its data rate while degrading the performance of the AU. Furthermore, the BD and AU compete for radio resources in competition symbiotic relationship. For example, when the AU transmits a signal, the BD backscatters a signal with a maximum reflection coefficient and causes interference. In response, the AU transmits at high power to improve its performance and suppress the weak BD signal. Through this relationship, both devices aim to improve their performance by harming each other. Despite these differences, the ultimate goal is to create a flexible SRad design for efficient resource sharing. The results presented in Figure 6 demonstrate that the BER performance of AU can be significantly improved in an SRad when BD cooperatively shares the resource and provides multipath. In particular, a performance gain of around 3 dB and 6 dB is achieved in AU and BD, respectively, when the ratio of SNR between backscattering link and direct link Δζ changes from −10 dB to 0 dB [22,48,65]. Moreover, the ratio of BD symbol to the AU symbol *N* also affects the BER performance. Figure 6a shows that increasing *N* from 20 to 40 enhances the BER of AU. This increase in the value of *N* also improves the performance of BD, as shown in Figure 6b.

To analyze the true potential of SRad, the authors [66] evaluate the SCm with commensal, parasitic, and competition relationships to increase the data rate of active and passive radio systems. In a commensal relationship, the AU endeavors to maximize its data rate without considering the performance of the passive radio system. Conversely, in a parasitic relationship, the BD transmits with a maximum reflection coefficient and at the same rate as the active user to achieve the maximum data rate, while sacrificing the transmission rate of the active radio system. Both systems strive to attain the maximum transmission rate by competing for resources, thus harming each other in the competition. Besides, in commensal and parasitic symbiotic relations, the spectrum growth problem occurs because BD transmits at different data rates compared to an AU. This problem is solved by jointly optimizing the reflection coefficient and transmit power of BD and AU [67]. In [23], the authors apply optimal beamforming at the AT to find the optimal achievable rates of each system in both symbiotic relationships.

Combining the benefits of BackCom and CR in an SRad system can improve spectrum sharing and energy efficiency through SCm. In this approach, the secondary transmitter acts as a BD and reflects signals from the primary transmitter to send messages to the secondary receiver. This allows the secondary system to share not only the radio spectrum but also power and infrastructure with the primary system. Additionally, the secondary transmission provides multipath diversity to the primary system. The authors in [48] explore mutualistic spectrum sharing in an SRad system with CR-based ABCm and utilize joint decoding at the secondary receiver to achieve highly reliable BackCom. To further enhance performance, a full-duplex function is added to the primary transmitter and an RIS is placed in the environment to benefit both primary and secondary systems. In [68], the authors study the power resource optimization problem under hardware impairments constraints in SRad system with CR based active radio system and full-duplex BD. The problem is solved by maximizing both active radio system link rates subject to the CR link and sum rate.

### 5.2. Resource Allocation and Multiple Access

In an SRad system, radio systems may consist of multiple transmitters and receivers sharing the resources in a symbiotic manner. Interference between the users and resource allocation such as spectrum, time, and power are critical issues in the concurrent communication of multiple users. The energy-efficient power allocation for cooperative and non-cooperative SRad systems is studied in [69]. In particular, finite blocklength channel codes are exploited under different transmission rates and symbol periods of BD to minimize the transmission power of AT and maximize the energy efficiency of BD. The authors in [70] propose a scheduling system called timing SRad to minimize energy consumption and increase energy efficiency while ensuring a minimum required throughput for BD. This scheme allows BD to harvest energy from ambient signals and simultaneously send their information without interfering with each other by using the carrier of AU signal. The authors use mathematical methods called conic quadratic representation and sequential quadratic techniques to solve the non-convex optimization problem.

In a symbiotic radio network, multiple access refers to the capability of several AU and BD to simultaneously use the communication resources. It is crucial to ensure the effective use of communication resources and prevent interference between active and passive users by separating them in time, frequency, space, code, and power. In [71] authors investigate the interference issues in a multi-BD BackCom based SRad system, in which multiple BD transmit simultaneously to the AU. The authors designed the coding algorithms with orthogonal code chips to enable interference-free communication. A distributive multiple BD access scheme is proposed in [72] to overcome the frequent inter-system and inter-BD coordination. Precoding is performed at each BD by multiplying the data with random spreading code. Furthermore, an iterative algorithm is proposed to optimize the SINR at BD and minimize the transmission power of the active radio transmitter. A stochastic transceiver design is proposed in [73] to solve interference issues such as inter-BD and interference in the downlink transmission. A batch stochastic parallel decomposition algorithm is proposed to solve stochastic multiple ratios fractional non-convex problems such as coverage analysis and acquiring the real-time tag’s symbol information with minimum feedback signaling. The authors in [74] try to improve the energy efficiency of an active radio system in a multiple BD SRad system. The resource to BD are allocated based on a time-domain multiple access approach, and it is analyzed that the maximum energy efficiency is achieved when the maximum time is allocated to the BD with maximum throughput. In [75], the authors demonstrate a full mutualism relationship with a massive number of BD by separating them in the power domain to enhance the sum rate of the SRad system.

The non-orthogonal spectrum sharing between active and passive radio systems is also investigated in SRad system, in which two users share the same spectrum with separability in power, code, or space domains [76]. Different from the conventional NOMA schemes, the spectrum sharing between active and passive radio systems is inherently supported by SRad because the power of the backscattered signal is very low compared to that of the AU. Some studies have exploited NOMA to enhance the spectral efficiency and security of both active and passive radio systems [76,77,78,79]. In [76], an SRad system is proposed to enhance the ergodic rates of NOMA assisted BackCom system. Active radio system employs power domain NOMA between the near and far users while BD backscatters its signal to the near AU. To extract the data, the near AU applies successive interference cancellation and the far AU treats the BD signal as noise. A NOMA enhanced dynamic time division multiple access scheme is proposed in [77] to increase the spectral efficiency in an SRad system. Full-duplex functionalities are also adopted at the active radio transmitter to send the signals to the receiver while receiving backscatter signals from BD. An SRad with cellular-NOMA is proposed in [78], and the system performance is analyzed in terms of the outage and ergodic rates of cellular users and BD. A NOMA based decode and forward relay assisted SRad is investigated in [79]. The relay is considered to transmit information and power simultaneously. By employing the power domain NOMA, the relay assists in forwarding the information of an AU and parasite BD to their destinations. While observing the performance of both systems, it is concluded that this relaying mechanism achieves better throughput than the conventional one at the cost of minor throughput degradation of AU.

### 5.3. MIMO and Beamforming

**Figure 7:**
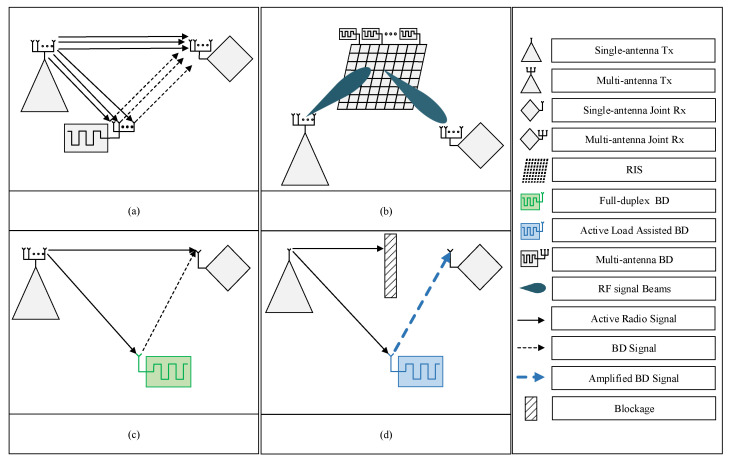
Scenarios of SRad with integration of advanced technologies (**a**) MIMO (**b**) RIS (**c**) full-duplex BD (**d**) active-load assisted BD.

MIMO technology allows the use of multiple antennas to simultaneously transmit and receive multiple data streams, increasing network capacity and reliability. This reduces vulnerability to fading and other channel impairments and mitigates interference through beamforming techniques [80]. MIMO improves energy efficiency by reducing power consumption, leading to a more efficient and reliable SRad system with higher capacity, better coverage, and improved energy efficiency. Different studies have been conducted to exploit the benefits of MIMO and beamforming in SRad systems, [81,82,83,84,85]. An end-to-end MIMO SRad system is proposed in [81], where each component of the system is equipped with multiple antennas as shown in Figure 7a. An optimal beamforming design problem is investigated to maximize the transmission rate of BD subject to the constraints of AT transmission rates. To solve this problem, a locally optimal solution is proposed based on an exact penalty to obtain the capacity upper bound. An SRad system with cell-free massive MIMO is investigated in [82]. Firstly, a two-step uplink training algorithm is proposed to estimate the CSI of the backscattered and direct links, wherein the first step is to estimate the direct link from the pilots received from AT without the involvement of BD while in the second step, both BD and AT transmit for estimation of backscattered link. Secondly, low complex beamforming is exploited to derive the achievable transmission rates of both systems. The authors in [83] focus on the limitations of active radio link enhancement by single BD, and how the full mutualism of symbiotic radio can be achieved by using MIMO SRad systems with massive BD. They derive the achievable rates of primary active and secondary passive communication and consider the asymptotic regime as the number of BD increases. They also study the precoding optimization problem to maximize the AU rate while ensuring that the BD communication rate is no lower than a certain threshold. Moreover, the authors in [84] propose a beam selection scheme to improve access of BD to RF signals in a millimeter wave SRad system. As the millimeter-wave channel is sparse, it is possible that some of the BD lack RF signals. The scheme selects beams based on channel measurement and dominant paths between AT and AU, ensuring signal accessibility to BD, and leading to an improvement in the overall system’s sum rate.

On the other hand, beamforming can help in securing the SRad systems from eavesdropping at the physical layer. The authors in [85], propose a secure beamforming approach to enhance the secrecy of BD in a multiple input-single-output NOMA-based SRad system. They assume the presence of a potential eavesdropper in the network and maximize the secrecy rate with a constrained concave-convex procedure-based algorithm from the BD to the central AU while considering the achievable secrecy rate constraints from the AT to the central and cell-edge AU. Moreover, beamforming can be used to create the null in the direction of the eavesdropper to prevent her from listening to the SCm and ensuring the privacy of users. Although MIMO and beamforming techniques are applied to maximize the transmission, and secrecy rate of SRad systems, there are major challenges to overcome such as accurate channel estimation, synchronization, and efficient spatial resource allocation. Besides, the use of multiple antennas and beamforming techniques at BD leads to high computational cost and power consumption, which can be a problem for passive IoT devices. To address this, new techniques are needed to reduce the complexity of the MIMO for SRad system and improve energy efficiency.

### 5.4. Reconfigurable Intelligent Surface Assistance

LIS or RIS is a two-dimensional metasurface that consists of a large number of low-power scattering components. It is capable of reflecting the incident signals into desired directions through passive beamforming [86,87]. Recently, due to its low power consumption and passive beamforming feature, it has been considered a promising solution to minimize the transmit power active radio system while enhancing the achievable rate of BD in a SRad system [88,89,90,91,92]. A simple RIS assisted SRad is shown in Figure 7b, where multiple BD are integrated to the RIS and passive beamforming is approach is used to improve the communication link of AU and to backscatter the information of BD to the BackCom receiver.

RIS is also deployed in a downlink SRad system to enhance the performance of active radio system and multi-BD BackCom system in [88]. The transmission power of the active radio system is minimized by jointly designing the beamforming at AT and RIS considering the SNR and transmission rates of both systems as optimization constraints. A cooperative beamforming approach is proposed in [89] for a simple BackCom based SRad system. RIS transmits the data of BD (i.e., connected to it through a wire) to a joint receiver by modulating the incident signals while configuring those signals to enhance the performance of AU. RIS can also transmit the data of multiple BD connected to it via wired links to the BackCom receiver while improving the wireless channel of the AU. A joint optimization approach for active beamforming at radio transmitter and passive beamforming at LIS is studied in [90]. The aim is to enhance the transmission rate of the passive radio system without affecting the rate of the active radio system. Other works on RIS assisted SRad system with multiple AU and a single BD are also investigated in the literature [91,92]. As RIS reflects the signals to an AU, it simultaneously modulates the data of BD. This study focuses on the power minimization problem under the rate constraints of AU and BD. The problem is solved by jointly optimizing active and passive beamforming at AT and RIS, respectively. Implementation of RIS in SRad can provide several benefits, such as improving the performance of an active radio system while enabling multi-BD communication. Readers interested in RIS assisted enhancement in SRad for spectral efficiency and energy efficiency are referred to [93].

### 5.5. Full-Duplex Techniques

Full-duplex technology is critical for spectral efficiency and reducing the system’s latency. Full-duplexing at AT and BD can improve the data rate and resource utilization in SRad system. For instance, an SRad system with full-duplex BD is shown in Figure 7c which is capable of simultaneously transmitting to AU while receiving data from AT in the incident signal. In particular, the BD divides the incident signal into two parts, one for decoding the signal and the other for backscattering its data. To analyze the potential of this technology in SRad, a low complexity full-duplex BD design is proposed in [94]. The authors optimize transmit power at an AT while optimizing the power splitting factor at BD. The performance of the system is measured in terms of transmission rates of BD. The research on full-duplex communication is still in its infancy, and new studies are needed to understand its limits in SRad for enhancing spectral and energy efficiency.

### 5.6. Active-Load Assistance

One of the main reasons for the short range of BD transmission is double-fading attenuation. The backscatter signal suffers from strong interference due to the high power of direct path active radio signal [95]. One way to solve this problem is to use multiple antennas at BD similar to RIS and increase the signal power through passive beamforming [90]. However, this is not possible in all cases, especially when the BD is a small IoT device or sensor. In this case, an active load can be used at BD to amplify and backscatter the incident signal through negative resistance [96]. In [95], authors investigate an active load assisted SRad to increase the range of BackCom in CR network. Specifically, an active load is used in BD to amplify the backscattered signal using negative resistances, increasing the transmission range. Besides, the dynamic range between the backscatter and direct link signal powers is also reduced and the probability of correct signal detection is increased. However, BD with active load may create severe interference to the primary signal in a parasitic relationship. Therefore, a beamforming vector is designed to minimize the interference while maximizing the rate of BD. This approach requires additional power but still has a lower power consumption than an active radio or RF chains for beamforming. Additionally, it reduces the dynamic range between the direct-link signal power and the backscatter-link signal power, making it easier to recover the BD signal [97]. BD with active load can also act as a relay while backscattering its data in case of direct signal blockage at the AU [98]. An SRad system with active-load assisted BD is shown in Figure 7d, where the AU does not have a direct signal from an active radio transmitter due to blockage, but it can receive the signal from the BD. To assist AU, the BD amplifies the incident signal while modulating its data over the signal. Further research is needed to fully understand the potential of active load-assisted BD in enhancing the performance of passive radio systems. An exciting avenue for future investigation could be the integration of active load-assisted BDs with full-duplex technology and RIS in various symbiotic relationships. Table 1 provides a summary of the developments in SRad along with the classification in terms of symbiotic relationships.

## 6. Applications for 6G and Beyond

**Figure 8:**
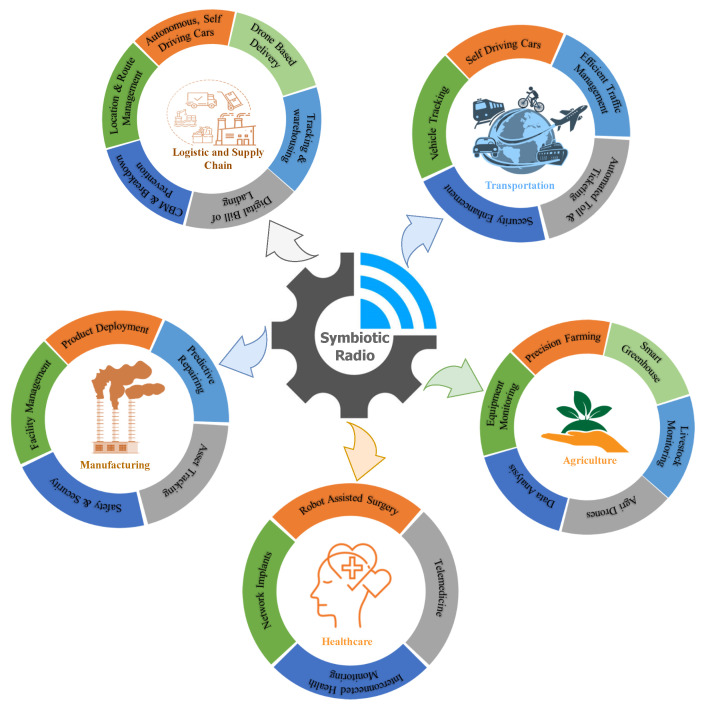
Applications of Symbiotic Radio.

The SRad can enable a wide range of applications in 6G and beyond. The existing and future wireless networks could aid power-constrained devices with symbiotic communication, particularly in locations with extensive passive sensors deployment. This section will go through some of the potential application areas of SRad that are shown in Figure 8.

### 6.1. Healthcare and Living

6G technology is foreseen to bring advancements in the healthcare field through robot-assisted surgery, telemedicine, and deep body implants with interconnected devices. These medical facilities require continuous health data collection and transfer through wireless devices and sensors, primarily low-power, and battery-operated [100]. Active radios for wireless data transfer in medical devices significantly reduce battery life. Also, complex RF circuits and signal processing techniques limit their use in wearables and tiny deep implants [101,102]. Implementing BackCom in medical devices can permanently overcome power issues and enhance battery life. However, traditional BackCom methods need the signal from dedicated or ambient RF sources, resulting in high system costs and limited connectivity. Through SRad, medical devices can reliably use the signal of existing wireless systems. For instance, WiFi access points available in hospitals or homes can support these while serving their users. In return, WiFi users can benefit from the backscattered signals as multipath under mutualistic relationships for performance enhancement. Moreover, medical devices frequently transfer their information to a centralized or a cloud server that needs a gateway in the localized network. In SRad, medical devices could be able to transfer their information online through WiFi. Furthermore, outdoor medical devices can connect to a cellular base station for SCm via commensal or mutual relationship and get the required radio resources.

The elderly population is increasing rapidly, and older people prefer to stay at home. Due to old age, people suffer from loneliness and diseases such as dementia, which creates difficulty for them to locate their daily use things, e.g., medicine kits. SRad based passive IoT devices can assist the older person in their daily activities at home or outside by locating personal belongings and providing environmental information. The household devices equipped with passive sensors can send their data to the cell phone with SCm utilizing WiFi network resources in the uplink/downlink [103]. WiFi nodes, in return, can get some helpful information from the passive devices about the environment to improve its quality of service working in different symbiotic relationships.

### 6.2. Agriculture

Collecting information about the environment and utilizing it to support agriculture is realized through an IoT-based ecosystem. IoT devices and sensors are placed on plants for precision farming and data gathering, particularly in smart greenhouses. These devices should be small and power efficient for ultra-dense deployment and must operate for a long time. Existing communication technologies for wireless sensor networks, e.g., Zigbee and Bluetooth, consume high power and are large. SRad can overcome these challenges by SCm while operating in a commensal relationship with TV and radio base stations. Moreover, the application of nano UAV and UGV in agriculture for online surveillance of fruit trees/crops and soil measurements has gained popularity [104]. These tiny unmanned devices communicate with other UAV and UGV through power-consuming radios that reduce their active duration. Implementing SRad in these vehicles can greatly enhance their energy efficiency and operating time. Instead of communicating via active radios, they can leverage SCm with backscatter radios. Additionally, they can develop a symbiotic relationship with the available cellular or satellite network to attain an RF carrier.

### 6.3. Transportation

ITS is evolving with the advancements in information and communication technologies. Autonomous driving, active collision protection, driver assistance, and mobility management systems are being developed to reduce road fatalities. These systems collect data from the environment through sensors and process it to take necessary actions. The collected data is also shared with nearby vehicles and the infrastructure using IEEE 802.11p and cellular V2X communication technologies. IoT sensors and devices in vehicular networks are battery-operated and must send active radio signals for information transfer. With the rapid expansion of transportation systems, a massive sensor deployment is expected globally, requiring more frequent data transfer. As a result, future ITS needs communication radios that are power-efficient and sophisticated in their utilization of radio resources. Strong research efforts are being conducted to replace active radios with backscatter radios. For instance, to improve driver’s awareness, information on pedestrians crossing the road was supplied to the car with a passive sensor utilizing a BackCom radio in [105]. SRad can enhance ITS with SCm in symbiosis with cellular V2X and IEEE 802.11p networks to encourage spectrum and energy-efficient communication.

### 6.4. Manufacturing

SRad technology holds the potential to revolutionize the manufacturing industry by improving the efficiency, productivity, and scalability of processes. The deployment of industrial IoT devices enabled by SRad technology can allow for real-time monitoring and control of manufacturing operations [106]. This includes the ability to perform predictive maintenance to detect equipment failures early, automation for improved efficiency and reduced labor costs, and smart factory implementation for real-time communication between machines. Additionally, SRad technology can be used for asset tracking to improve inventory management and supply chain efficiency. The symbiotic relationships between different radio systems with SRad technology enable the integration of different manufacturing processes into a cohesive system, leading to improved efficiency and scalability. Furthermore, SRad technology can be utilized for monitoring and control of safety-critical systems, ensuring the safety of employees in the manufacturing facility. Overall, SRad technology has the potential to play a key role in the digitalization and automation of the manufacturing industry, enabling efficient, smart, and sustainable manufacturing.

### 6.5. Logistic and Supply Chain

SRad technology can be used in logistics and supply chain management to improve communication and efficiency. Usually, RFID tags are connected to the products for tracking and management [107]. Manual scanning of a large number of tags is a time-consuming task and requires human effort. SRad can be used for real-time asset location tracking and inventory management in a warehouse, by using available WiFi access points to locate and manage inventory. RFID tags can be replaced with passive IoT devices that modulate the signals of WiFi, and it gets their information and location from the received signal, reducing the need for manual scanning. Additionally, SRad can be used for real-time shipment tracking outdoors by using the cellular network. By intelligently allocating and adapting the available spectrum, SRad can enable real-time communication and coordination, reducing delays and costs and improving the overall performance of logistics and supply chain operations. This can provide better visibility and control over the supply chain, reducing delays and increasing efficiency. Additionally, it can improve RFID tagging by minimizing interference and optimizing communication range, resulting in improved accuracy and reliability of RFID data. Fleet management can also be improved by enabling real-time tracking and coordination, reducing delays, and increasing efficiency. Furthermore, warehouse management can be improved by enabling real-time tracking of inventory, optimizing communication, and reducing the time required to locate and retrieve items.

## 7. Open Problems and Future Directions

Given the existing research advancements in the field of SRad, various challenges call for attention to pave the way for future research directions. Addressing these challenges is essential to enhancing the efficiency of SRad, in terms of resource allocation, improving the coexistence of active and passive radio systems, and simplifying decision-making processes within SRad networks. This section highlights the problems and outlines the promising avenues for future research in SRad.

### 7.1. Channel Modelling and Estimation

Accurately modeling and estimating the channel between the different nodes in the network are the critical challenges in SRad system. The channel in SRad system is typically composed of both active and passive links, making it difficult to estimate accurately. The active links are formed by the traditional wireless communication between the AU and their corresponding user equipment, while the passive links are formed by the BackCom between the BD and the AT. The CSI of both the active and passive links is required for efficient resource allocation and signal processing in SRad system. In the literature, various channel estimation techniques have been proposed for SRad systems. For example, in [108], the authors propose an expectation maximization algorithm to design a blind channel estimator for acquiring channel parameters. In [82], a novel two-phase uplink-training-based channel estimation scheme is proposed for cell-free SR systems, which is based on the linear minimum mean square error estimation. The authors in [109] use deep learning to design for joint pilot design and channel estimation in SRad systems. The interference and noise between different channels are canceled through successive interference cancellation and deep residual neural networks. However, these channel estimation schemes typically face challenges such as high computational complexity and the need for linear optimization. It is essential to investigate and improve channel estimation techniques to enhance the performance and energy efficiency of SRad systems. This can be achieved by developing new channel estimation algorithms that are more computationally efficient and by incorporating machine learning techniques to better model the SRad channel.

### 7.2. Synchronization Challenges

In an SRad system, the signal transmission of two systems can be sporadic. BD needs the incident RF signal every time there are data to be transmitted. In case of signal unavailability, the BD must wait until AT transmits the signal, which causes a delay in the transmission. In critical applications such as healthcare and transportation, the delay in transmission can cause severe damage. One promising solution is to enable synchronization between BD and AT for the pre-knowledge of signal transmission time. However, it is challenging in an SRad system due to the low processing capabilities of BD. The circuits used in AU for timing and phase recovery are power-consuming and unsuitable for low-power devices. Also, the synchronization methods developed for active radio transceivers are inapplicable to passive devices due to hardware and power limitations. Although MIMO and active load assisted BD are used for synchronization, their power consumption and complexity constraints limit their applicability. Research can be directed to multi-antenna BD design for developing simple synchronization algorithms. Full-duplex BD can also be explored as it allows simultaneous energy harvesting and data detection from the received signal.

### 7.3. Downlink/Uplink Feedback

The low power and simple processing constraints limit the capabilities of the BD as a receiver. However, the downlink control channel is necessary to design a physical layer protocol for an SRad system. Besides, the uplink channel design is also crucial for feedback and data transfer. A non-coherent receiver design is comparatively simpler than the coherent one, but its performance is low. Moreover, an energy detector requires a much larger symbol length of BD signal than an active radio signal to average out the underlying modulated signal. Developing a new low-processing downlink signaling/feedback mechanism is a promising research direction.

### 7.4. Interference Management

Managing interference between radio systems in parasitic or competitive relationships is a complex task. In general, the direct signal from the AT causes significant interference to the BD signal at the receiver. However, the BD signal can also interfere with the AU in both commensal and competitive relationships during uplink transmission. Furthermore, intra-BD interference may occur when multiple BD are communicating over the same carrier signal in an SRad system. This type of interference can lead to a higher packet error rate. To reduce the error rate, various methods can be employed such as successive interference cancellation and beamforming. In the successive interference cancellation method, the direct path signal must be eliminated before decoding the backscattered signal. In contrast, the beamforming approach involves directing the antenna’s null towards the direction of the interfering signal, followed by sampling the weak backscattered signal using a traditional analog-to-digital converter and detecting it using a non-coherent receiver. Additionally, coding and robust signal processing techniques can be implemented at the BD to further improve performance.

### 7.5. Multi-Antenna Design Problems

Multi-antenna technology and its implementation protocols and algorithms have been explored extensively in active radio systems but they are not directly applicable to passive radio systems due to high power consumption and complexity. Nowadays, multiple antenna BD with beamforming are considered for improving achievable rates of active and passive radios in SRad systems. A hybrid beamforming scheme for SRad system with mmWave and passive IoT system is investigated in [110], where the authors perform active beamforming at AU and passive beamforming at BD to achieve high data rates. However, the proposed beamforming mechanisms are computationally complex and impractical for low-power IoT devices. New low-complex signal processing algorithms and passive beamforming techniques are needed for SCm. Besides, joint beamforming strategies can also be explored in the mutualistic SRad system.

### 7.6. Network Design and Management Issues

SRad systems involve the integration of active and passive devices due to which a number of unique challenges arise in terms of network design and management. One major issue is the selection of the appropriate RF source in a heterogenous network to achieve the desired balance between energy harvesting, backscattering, energy efficiency, and data rates. This can be further complicated by the fact that the propagation and availability of ambient signals can vary significantly depending on the environment, making it difficult to predict the best RF source for a given scenario. Another key challenge is the management of multiple AU and BD in the system. This requires real-time channel estimation of all active and passive users, which can be challenging to achieve in practice. Additionally, there is the problem of determining the optimal user association to maximize the overall system sum rate. Scalability is also an important consideration in the design of symbiotic radio systems, as the number of BD can vary significantly depending on the environment. This can make it difficult to develop effective resource allocation and management strategies that can adapt to changing network conditions. In [99], the authors present two deep learning methods, CDRL and DDRL, to infer the current CSI from past CSI. Both methods achieve optimal AU and BD association, which is close to the one with perfect real-time CSI. Furthermore, in terms of scalability for a large number of BD, the DDRL method surpasses the CDRL approach. CDRL, on the other hand, requires less information than the DDRL to solve the association issue. However, the use of deep learning methods for user association in an SRad system with heterogeneous networks is an open area of research. Overall, the network design and management issues in SRad systems are complex and multifaceted. Further research is needed to develop effective solutions that can address these challenges and enable the successful deployment of SRad systems in real-world scenarios.

### 7.7. Security and Privacy Concerns

The sharing of radio resources between different radio systems and the broadcast nature of passive devices makes the SRad vulnerable to attacks. Common types of attacks include eavesdropping, jamming, and spoofing [111]. For instance, an illegitimate user in the network can exploit the broadcast nature of BD to listen to their sensitive information by eavesdropping. It can also disrupt the SCm by jamming the signal of AT so that the AU and BD can not communicate in SRad system. Besides in a spoofing attack, a malicious AU in the network can emulate intentionally as a legitimate receiver to get the data of BD in passive IoT. It is essential to secure the design of low-complexity security algorithms to secure passive devices in the SRad system. The conventional cryptography-based encryption and authentication methods designed for active radio systems are inapplicable in processing-restricted BD due to their higher computational complexity and key management issues [112].

On the other hand, physical layer security is a promising solution for providing security and privacy leveraging by leveraging the dynamic aspects of the wireless environment [113]. Different approaches can be used to secure the SRad system at the physical layer. The authentication can be performed by exploiting the RF fingerprint BD and AU as each one of them has a unique RF fingerprint due to hardware impairments. The active jamming or beam nulling techniques can be exploited to prevent malicious BD from using the radio resources and creating interference in the network. Besides, artificial noise can be added to the carrier signal for covert SCm. The artificial noise can be added at AT before transmission of the carrier signal or AU can transmit an artificial noise signal that is known to the receiver during the BackCom [114]. These techniques are suitable for securing single BD from security attacks but not scalable to the network level where multiple BD and AU perform concurrent transmission. It is an urgent need to develop scalable physical layer security techniques, especially for jamming and eavesdropping. Other than that, in a SRad system with heterogeneous networks, it is vital to ensure that only authorized devices have access to shared radio resources.

### 7.8. Defining Key Performance Metrics

In an SRad system with a commensal symbiotic relationship, BD can provide multipath diversity to AU in order to increase their reliability. By utilizing a mutualism symbiotic relationship, the overall capacity of the system can also be increased by jointly increasing the sum rate. Investigating new methods for spectral efficiency enhancement through SRad with various symbiotic relationships between active and passive radio systems is an exciting research area to be explored. Moreover, in the SRad system, radio resources are shared across multiple dimensions, such as spectrum, energy, and infrastructure. These new forms of resource sharing require novel performance metrics to accurately measure system performance, as opposed to traditional metrics.

### 7.9. Symbiotic Communication and Sensing

SRad can be designed not only for communication but also for sensing in an obligatory symbiosis which leads to the idea of SCAS. To enable SCAS, a passive radio system can coexist with an active radio system that performs joint Radar/sensing and communication. The signal transmitted for cooperative sensing and communication can also be utilized by BD to backscatter its information. Then, the active radio transceiver can detect the data of BD while performing sensing and communication.

**Figure 9:**
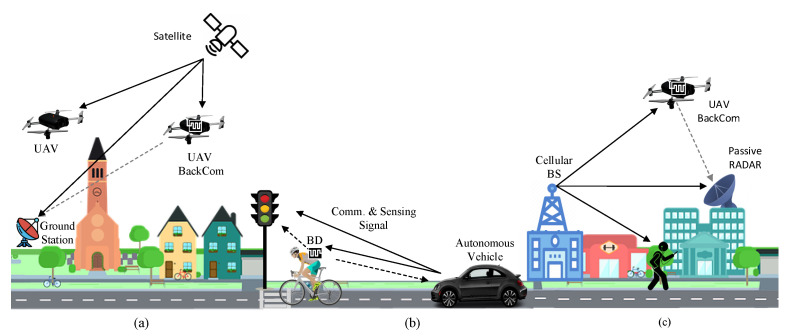
Symbiotic communication and sensing use cases; (**a**) non-terrestrial networks (**b**) vehicle-to-everything, (**c**) passive Radar system.

In [115], a frequency-modulated continuous wave signal is used to perform localization and BackCom. The authors also propose a framework for joint processing of BackCom and Radar signals and a line coding-based filter for clutter removal to detect the target of interest within clutter return. In [116], the author proposes an extension of this work for distributed sensing and communication. We anticipate three use cases of SCAS, including non-terrestrial networks, V2X, and passive Radar. Figure 9a shows air-to-air and air-to-ground SCAS scenarios in a non-terrestrial network. A satellite transmits a communication signal to UAV and a ground station. The UAV backscatters the signal to send its data with BackCom, and when the ground station receives the backscattered signal it performs SCAS to find the location of UAV along with the data. In contrast, Figure 9b illustrates SCAS for autonomous driving in V2X, where an autonomous vehicle transmits the frequency-modulated continuous wave signal to sense the environment and communicates with traffic light control system while an IoT device e.g., smartwatch, attached to cyclist sends the data to the vehicle through BackCom. Figure 9c shows the third application of SCAS in a passive Radar system, in which the passive Radar uses the signal of the cellular base station to detect the presence of a UAV and also receive its information through transmitted by BackCom. The SRad technology for SCAS is still in its infancy, and future research can be focused on SCAS waveform design, channel estimation through sensing signal, joint Radar and sensing parameter extraction, and backscattered signal interference cancellation from joint communication and sensing signal at the receiver. The development of SRad system for SCAS can enable new paradigms for spectrum sharing and the coexistence of multiple radio systems with individual functionalities.

## 8. Conclusions

This article presents a comprehensive literature review on SRad technology as a new paradigm for resource sharing and management between passive and active radio systems. We started by classifying radio systems and introduced fundamental concepts of SRad. Then, symbiotic relationships with varying realizations, ranging from mutualism to competition, are explored for efficient resource sharing. Subsequently, we reviewed the state-of-the-art methodologies in SRad systems, including SCm, resource allocation, and multiple access. The potential applications of SRad in 6G and beyond for various domains such as healthcare, transportation, manufacturing, supply chain, and agriculture are also discussed from a deployment perspective. Additionally, we highlighted critical challenges in SRad systems related to security, channel modeling, network design, interference management, and more. An SCAS paradigm of SRad is also explained along with its use cases in non-terrestrial networks, V2X, and passive radar systems. It has been noticed that the performance and deployment of SRad may be limited by its implementation complexity, lack of standard technical specifications and protocols, interference and security concerns. Despite these shortcomings, we foresee SRad as a potential candidate for efficient resource sharing and coordination between dissimilar radio systems in the future.

## Figures and Tables

**Table 1:** Overview of SRad system developments.

Reference	Design Objective	System	Symbiotic Relationships			
			Mutualistic	Commensal	Parasitic	Competitive
[23]	Rate maximization of BD and power minimization at active transmitter	Single BD	🗸	**–**	**–**	**–**
[48]	Rate maximization of passive and active radio system	Single BD	🗸	**–**	**–**	**–**
[66]	Rate maximization of passive and active radio system	Single BD	**–**	🗸	🗸	🗸
[67]	Resource allocation under fading channels	Single BD	**–**	🗸	🗸	**–**
[68]	Power optimization of CR based active radio system under hardware impairments	Single BD	🗸	🗸	**–**	**–**
[69]	Resource allocation with cooperative and non-cooperative active radio system	Single BD	**–**	🗸	🗸	**–**
[70]	Optimal resource allocation by scheduling BD in time	Multi-BD	**–**	🗸	**–**	**–**
[71]	Random access with orthogonal interference	Multi-BD	**–**	🗸	**–**	**–**
[72]	Power optimization in random code based multiple access	Multi-BD	**–**	**–**	🗸	**–**
[73]	Stochastic transceiver design for performance improvement under downlink and inter-BD interference	Multi-BD	**–**	**–**	🗸	**–**
[74]	Resource allocation to maximize energy efficiency of active radio system	Multi-BD	🗸	**–**	**–**	**–**
[75]	Sum rate maximization of active users with massive number of BD	Multi-BD	**–**	🗸	🗸	**–**
[76]	Outage probability and ergodic rate analysis of backscatter NOMA	Single BD	**–**	🗸	**–**	**–**
[77]	Throughput maximization of BD in NOMA-DTDMA system	Multi-BD	**–**	**–**	🗸	**–**
[78]	Outage analysis under signal interference of cellular-NOMA AU and BD	Multi-BD	**–**	🗸	**–**	**–**
[79]	Performance analysis of decode and forward relay network	Multi-BD	**–**	**–**	🗸	**–**
[81]	BD transmission rate optimization	Multi-BD	**–**	**–**	🗸	**–**
[83]	AU link enhancement with massive BD deployment and MIMO receiver	Multi-BD	🗸	**–**	**–**	**–**
[85]	Secure beamforming and secrecy rate analysis of BD	Single BD	**–**	🗸	**–**	**–**
[84]	Signal accessibility to BD in millimeter wave beamspace channel through beam selection	Multi-BD	**–**	🗸	**–**	**–**
[88]	Joint transmit beamforming for power minimization of active radio system	Multi-BD	🗸	**–**	**–**	**–**
[89]	Transmit power minimization of active radio system	Single BD	**–**	🗸	**–**	**–**
[90]	Beamforming for BD rate enhancement	Multi-BD	🗸	**–**	**–**	**–**
[91]	Transmit power minimization of active radio system	Single BD	🗸	**–**	**–**	**–**
[92]	BD transmission rate enhancement and AT transmit power minimization	Single BD	🗸	**–**	**–**	**–**
[95]	BD transmission rate maximization with active load and interference reduction at AU with beamforming	Single BD	**–**	**–**	🗸	**–**
[99]	Optimal BD association to active users using deep learning methods	Multi-BD	**–**	**–**	🗸	**–**
[94]	Transmit power optimization of active radio system with full-duplex BD	Multi-BD	**–**	**–**	🗸	**–**

## Data Availability

Not applicable.

## Abbreviations

The following abbreviations are used in this manuscript:

3GPP	third generation partnership project
6G	sixth-generation
AmBackCom	ambient backscatter communication
AT	active transmitter
AU	active user
BackCom	backscatter communication
BD	backscatter device
BER	bit error rate
CDRL	centralized deep reinforcement learning
CR	cognitive radio
CSI	channel-state-information
DDRL	distributed deep reinforcement learning
DSA	dynamic spectrum access
FCC	Federal Communication Commission
IoT	Internet-of-Things
ISM	Industrial Scientific and Medical
ITS	intelligent transportation system
LIS	large-intelligent surface
LTE	long-term-evolution
MIMO	multiple-input multiple-output
mmWave	millimeter wave
NOMA	non-orthogonal multiple access
RADAR	radio detection and ranging
RF	radio frequency
RFID	radio frequency identification
RIS	reconfigurable intelligent surfaces
SCaS	symbiotic communication and sensing
SCm	symbiotic communication
SINR	signal-to-interference-plus-noise-ratio
SNR	signal-to-noise-ratio
SRad	symbiotic radio
SSA	static spectrum access
THz	terahertz
TV	television
TVWS	TV white space
UAV	unmanned aerial vehicle
UGV	unmanned ground vehicles
V2X	vehicle-to-everything
WiFi	wireless fidelity
